# Detecting ferroptosis and immune infiltration profiles in multiple system atrophy using postmortem brain tissue

**DOI:** 10.3389/fnins.2023.1269996

**Published:** 2023-12-29

**Authors:** Linxi Chen, Lingqun Mao, Hongsheng Lu, Peng Liu

**Affiliations:** ^1^Department of Neurology, Taizhou Central Hospital (Taizhou University Hospital), Taizhou, Zhejiang, China; ^2^Department of Pathology, Taizhou Central Hospital (Taizhou University Hospital), Taizhou, Zhejiang, China

**Keywords:** multiple system atrophy, ferroptosis, immune infiltration, α-synucleinopathy, RNA sequencing

## Abstract

**Background:**

The importance of ferroptosis and the immune system has been mentioned in the pathogenesis of α-synucleinopathy. The α-synuclein-immunoreactive inclusions that primarily affect oligodendrocytes are the hallmark of multiple system atrophy (MSA). Limited evidence implicates that iron and immune responses are involved in the pathogenesis of MSA, which is associated with neurodegeneration and α-synuclein aggregation.

**Methods:**

The RNA sequencing data were collected from the Gene Expression Omnibus database. MSA-C-related module genes were identified through weighted gene co-expression network analysis. Gene Ontology and Kyoto Encyclopedia of Genes and Genomes analyses were performed to predict the potential molecular functions. The candidate ferroptosis-related genes associated with MSA-C were obtained using a machine-learning algorithm. CIBERSORT was used to estimate the compositional patterns of the 22 types of immune cells.

**Results:**

The tissues for sequencing were extracted from postmortem cerebellar white matter tissues of 11 MSA-C patients and 47 healthy controls. The diagnostic ability of the six MSA-C-related ferroptosis-related genes in discriminating MSA-C from the healthy controls demonstrated a favorable diagnostic value, with the AUC ranging from 0.662 to 0.791. The proportion of CD8^+^ T cells in MSA-C was significantly higher than in the controls (*P* = 0.02). The proportion of NK cells resting in MSA-C was significantly higher than in the controls (*P* = 0.011).

**Conclusion:**

Ferroptosis and T-cell infiltration may be important pathways of disease development in MSA-C, and targeting therapies for these pathways may be disease-modifying.

## Introduction

Multiple system atrophy (MSA) is a sporadic, adult-onset, progressive neurodegenerative disease characterized by autonomic failure, cerebellar ataxia, and Parkinsonism (Gilman et al., 2008; Wenning et al., 2022). According to the clinical manifestations, MSA is classified into two categories: the predominant Parkinsonism subtype (MSA-P) and the predominant cerebellar ataxia subtype (MSA-C). The hallmark of MSA is the presence of α-synuclein-immunoreactive inclusions primarily affecting oligodendrocytes (Krismer and Wenning, 2017). MSA progresses rapidly, leading to high disability rates and reduced survival (Low et al., 2015). However, the underlying pathophysiological mechanism of MSA remains unclear, and effective treatments are lacking.

Iron is a vital element for the proper functioning of neurons and glial cells, playing critical roles in various metabolic processes. Ferroptosis, a recently discovered form of regulated cell death, is dependent on iron dyshomeostasis and lipid peroxidation (Tang and Kroemer, 2020; Ou et al., 2022). Ferroptotic cell death has been implicated in a growing list of pathophysiological processes and is linked to dysregulated immune responses (Chen et al., 2021). It has been suggested that ferroptosis is associated with neurodegenerative diseases, such as Parkinson's disease (PD), Alzheimer's disease, and Huntington's disease (Ou et al., 2022). Notably, oligodendrocytes are the cells with the highest iron content in the central nervous system (Kaindlstorfer et al., 2018). Limited evidence suggests that iron may be involved in the pathogenesis of MSA, a condition associated with neurodegeneration and α-synuclein aggregation in the context of oxidative stress, immune response, and neuroinflammation (Kaindlstorfer et al., 2018). However, the association between ferroptosis-related genes and MSA at the gene level remains unclear.

There is evidence to suggest that the innate immune system influences dopaminergic cell death (Allen Reish and Standaert, 2015). The importance of the immune system in the pathogenesis of α-synucleinopathy has been noted, with T-cell infiltration observed in the brains of individuals with PD (Brochard et al., 2009). Although research on MSA is scarce, evidence indicates an increase in pro-inflammatory cytokines in the cerebrospinal fluid and brain parenchyma of MSA patients, indicating the involvement of the immune system (Kaindlstorfer et al., 2018). The immune infiltration profile in the cerebellar tissue of MSA-C remains unknown and requires further investigation.

In order to explore the underlying pathophysiological mechanism in MSA-C, this study further analyzed the RNA sequencing data from cerebellar tissues to detect ferroptosis and immune infiltration profiles in MSA-C.

## Materials and methods

### Gene expression data

The RNA sequencing data (GSE199715) were collected from the Gene Expression Omnibus database (http://www.ncbi.nlm.nih.gov/geo). The tissues for sequencing were extracted from postmortem cerebellar white matter tissues of 11 MSA-C patients and 47 healthy controls (Table 1).

**Table 1:** Clinical features of samples.

	**MSA-C**	**HC**	***P*-value**
Number	11	47	
Female/male	4/7	31/16	0.093
Age at death (year)	63.55 ± 6.42	84.17 ± 9.09	0.000
Postmortem interval (hour)	56.85 ± 22.21	59.87 ± 28.23	0.742

MSA-C, multiple system atrophy with predominant cerebellar ataxia; HC, healthy control.

### Identification of MSA-c-related ferroptosis-related genes

Weighted gene co-expression network analysis (WGCNA) is a method for analyzing the gene expression patterns of multiple samples, which can cluster genes with similar expression patterns and analyze the association between modules and specific traits or phenotypes (Langfelder and Horvath, 2008). WGNCA was used to identify significant modules related to MSA-C. A total of 259 ferroptosis-related genes were collected from the FerrDb database (Zhou and Bao, 2020). MSA-C-related ferroptosis-related genes were obtained from the overlapping portion of MSA-C-related module genes and ferroptosis-related genes.

### Functional analysis

Gene ontology (GO) analyses, including biological processes, cellular components, and molecular functions, were performed to elucidate genetic regulatory networks of interest used to predict the potential molecular functions using the R package. The Kyoto Encyclopedia of Genes and Genomes (KEGG) analysis was used to predict the potential molecular functions using the R package.

### Machine learning for selecting candidate genes

The least absolute shrinkage and selection operator (LASSO) and support vector machine recursive feature elimination (SVM-RFE) were applied to select the candidate genes using the R package. LASSO is a regression analysis algorithm that uses regularization to improve prediction accuracy. The LASSO regression algorithm was carried out using the glmnet package in R. SVM-RFE aims to find a subset of features that results in an SVM model with the best possible classification performance. Mean misjudgment rates were computed and compared using 10-fold cross-validations. The overlapping genes between the two algorithms were further assessed for accuracy in diagnosing MSA-C patients using a receiver-operating characteristic (ROC) curve.

### Evaluation of the immune profile

CIBERSORT is an algorithm that uses gene expression data to estimate the abundance of member cell types in mixed-cell populations using the R package (Newman et al., 2015). In this study, we utilized a reference set comprising 22 immune cell genes (LM22). The number of permutations set was 1,000. The results with a *P* < 0.05 obtained from the CIBERSORT analysis were preserved. The correlation between various immune cells was assessed using Spearman's analysis. We further investigated the correlation between candidate genes and 22 immune cells.

### Statistical analysis

All analyses were performed using R software (version 4.1.3). Comparisons between the two groups were conducted using the independent *t*-test and Mann–Whitney *U*-test, as appropriate. Spearman's correlation was used to detect associations between parameters. Differences were regarded as statistically significant at a *P* < 0.05.

## Results

**Figure 1:**
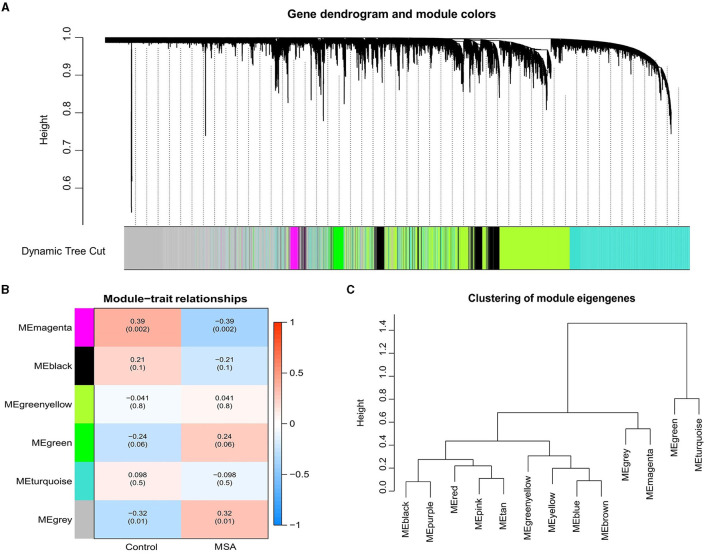
Weighted gene correlation network of the genes involved in MSA-C. **(A)** Gene clustering tree (dendrogram) obtained by hierarchical clustering based on adjacency-based dissimilarity. **(B)** Associations between clinical traits and modules. **(C)** Clustering of module eigengenes.

WGNCA was used to identify significant modules related to MSA-C. The clustering dendrogram identified six modules, of which the gray and magenta modules were significantly associated with the MSA-C phenotype (Cor > 0.3, *P* < 0.05, Figures 1A–C). A total of 4640 genes from these two modules were collected for further analysis, resulting in 61 MSA-C-related ferroptosis-related genes obtained from the overlapping portion of MSA-C-related module genes and ferroptosis-related genes.

**Figure 2:**
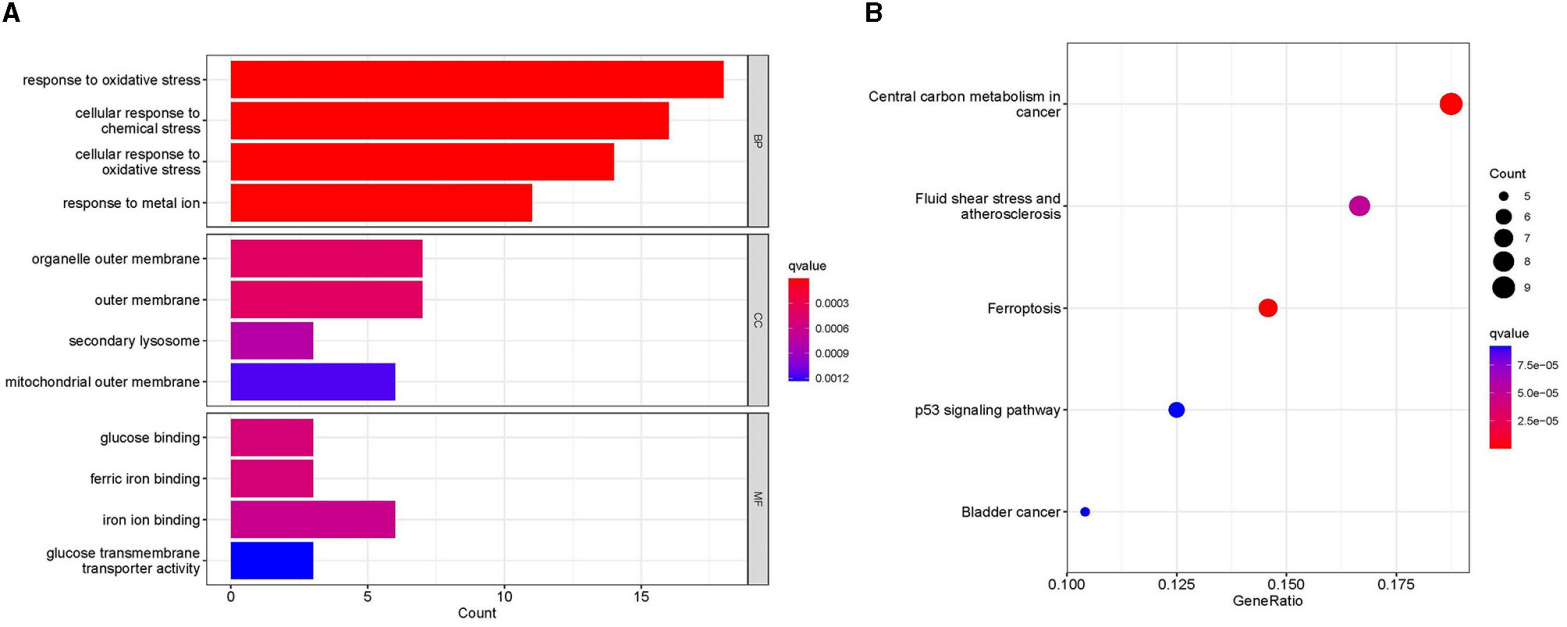
Functional analyses. **(A)** Gene ontology analysis, including biological process, cellular component, and molecular function, showed the enrichment of ferroptosis-related module genes. **(B)** Kyoto Encyclopedia of Genes and Genomes analysis showed the pathway enrichment of ferroptosis-related module genes.

The GO analysis of 61 MSA-C-related ferroptosis-related genes revealed that changes in biological processes were primarily focused on the response to stress (Figure 2A). The results of the cell component analysis showed enrichment in the organelle outer membrane, outer membrane, secondary lysosome, and mitochondrial outer membrane (Figure 2A). The most enriched molecular function annotations were glucose binding, ferric iron binding, iron ion binding, and glucose transmembrane transporter activity (Figure 2A). The KEGG analysis revealed that these overlapped genes were mainly enriched in pathways involved in central carbon metabolism in cancer, fluid shear stress and atherosclerosis, ferroptosis, and the p53 signaling pathway (Figure 2B).

**Figure 3:**
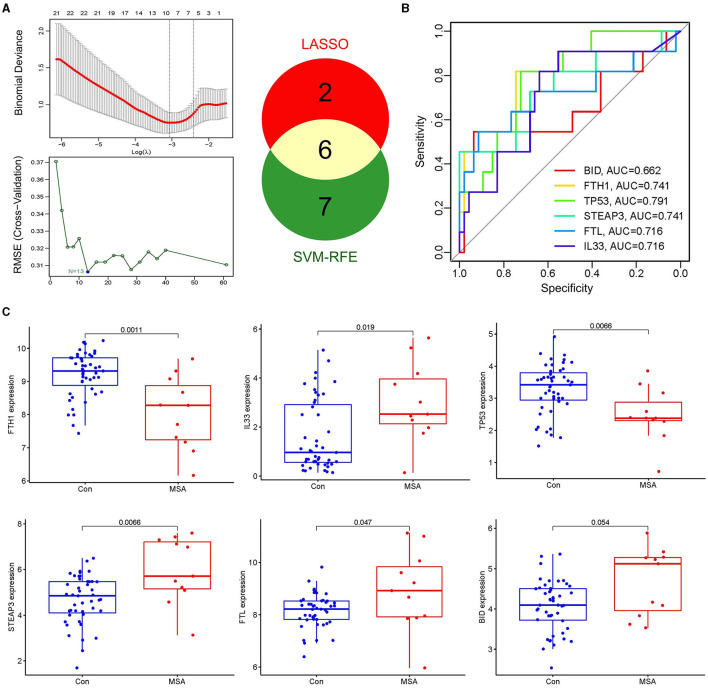
Selection of candidate genes and their expression level. **(A)** Eight features were selected by the LASSO algorithm, and 13 features were selected by the SVM-RFE algorithm. Six overlapping genes between the two algorithms, namely *FTH1, IL33, TP53, STEAP3, FTL*, and *BID*, were identified as the MSA-C-related ferroptosis-related genes. **(B)** Diagnostic ability of the six biomarkers in discriminating MSA-C from healthy controls. **(C)** Expression levels of candidate genes.

LASSO and SVM-RFE algorithms were applied to select candidate genes. Among the 61 ferroptosis-related genes in MSA-C, the LASSO algorithm selected 8 features, and the SVM-RFE algorithm selected 13 features (Figure 3A). Six genes, namely, *FTH1, IL33, TP53, STEAP3, FTL*, and *BID*, were identified as MSA-C-related ferroptosis-related genes through the overlapping genes between the two algorithms (Figure 3A). These six biomarkers exhibited favorable diagnostic value in discriminating MSA-C from healthy controls, with the area under the curve (AUC) ranging from 0.662 to 0.791 (Figure 3B). Expression levels of *FTH1* and *TP53* in the MSA-C tissue were significantly lower than those in healthy controls, whereas the expression levels of *IL33, STEAP3*, and *FTL* in the MSA-C tissue were significantly higher (Figure 3C).

**Figure 4:**
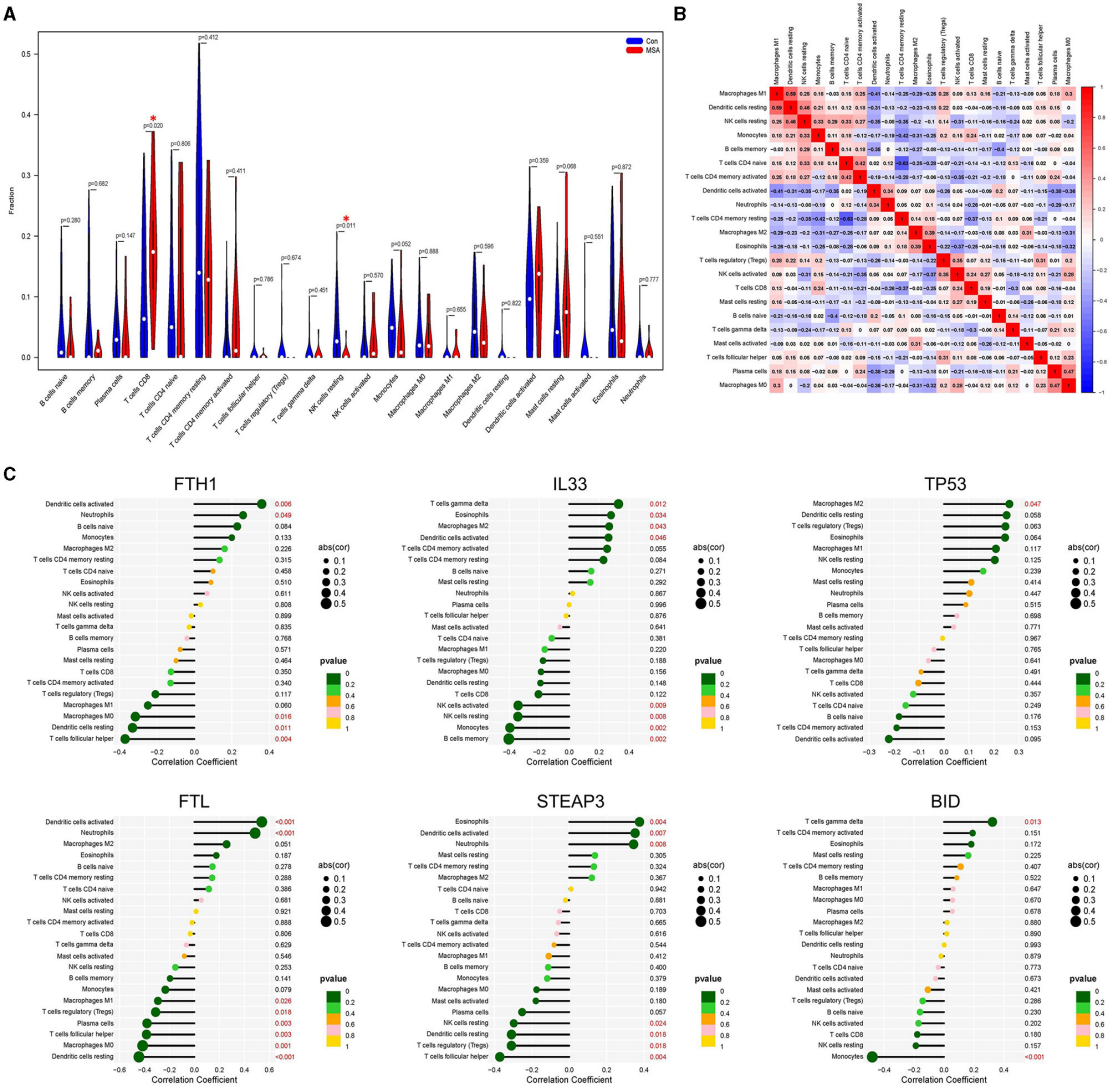
Evaluation of the immune profile. **(A)** Comparison of 22 immune cells between MSA-C tissues and healthy control tissues. **(B)** Heatmap showing the correlation matrix of all 22 immune cell compositions. **(C)** Correlation between ferroptosis-related candidate genes and immune cells.

The composition of immune cells in postmortem cerebellar white matter tissues of MSA-C patients and healthy controls was explored. The proportion of CD8^+^ T cells in MSA-C was significantly higher than in controls (*P* = 0.02, Figure 4A). Similarly, the proportion of resting NK cells in MSA-C was significantly higher than in controls (*P* = 0.011, Figure 4A). The correlation between 22 types of immune cells and the correlation between ferroptosis-related candidate genes and immune cells are shown in Figures 4B, C.

## Discussion

In this study, we employed a combination of WGCNA, machine learning, and CIBERSORT algorithms to analyze RNA sequencing data of the cerebellar white matter tissue from MSA-C patients and healthy controls. Our analysis revealed six ferroptosis-related genes associated with MSA-C, and we observed infiltration of CD8^+^ T cells in the cerebellar white matter of MSA-C patients. Additionally, correlation analysis demonstrated the association between these candidate ferroptosis-related genes and immune cells.

Oligodendrocytes, which are the richest iron-containing cells in the central nervous system, are responsible for myelination and metabolic enzyme activity (Kaindlstorfer et al., 2018; Wang et al., 2022). Additionally, oligodendrocytes are the primary source of aggregated α-synuclein in MSA (Meissner et al., 2019). Previous studies have shown diffuse ferritin deposition in the basal ganglia and cerebellar dentate nucleus in MSA, indicating a high iron content in degenerative brain regions (Matsusue et al., 2009). The regulatory role between iron and α-synuclein biology, including the ferroreductase activity of α-synuclein and iron-mediated translational regulation of ferroreductase, has led to the hypothesis that iron may play a role in MSA pathophysiology (Thomas and Jankovic, 2004; Berg and Hochstrasser, 2006; Rogers et al., 2011; Kaindlstorfer et al., 2018). The role of iron in MSA pathogenesis is complex and multifaceted. Excessive iron accumulation may lead to oxidative stress, mitochondrial dysfunction, and impaired autophagy, all of which are implicated in the pathogenesis of MSA (Jellinger, 2014). Additionally, iron overload may induce the production of reactive oxygen species, leading to DNA damage and neuronal death. The specific mechanism by which ferroptosis mediates MSA remain unclear, and further understanding of its molecular mechanisms may provide novel therapeutic targets for this devastating disease. In addition to its role in MSA, ferroptosis has been implicated in the pathogenesis of PD, another α-synucleinopathy. Previous studies have suggested that ferroptosis may contribute to α-synuclein aggregation and neuronal loss in PD (Devos et al., 2014; Martin-Bastida et al., 2017; Wang et al., 2022). Interestingly, ferritin heavy chain (*FTH1*), which encodes the heavy subunit of ferritin and is a major intracellular iron storage protein, has been reported as a potential contributing factor in PD (Tian et al., 2020; Li et al., 2021). *FTH1* exhibits ferroxidase activity through glutamate residues that act as metal ligands and promote rapid iron uptake (Levi et al., 1988; Wade et al., 1991; Cozzi et al., 2000). The expression of *FTH1* is significantly downregulated in 6-hydroyxdopamine-induced rat and PC-12 cell models of PD, and *FTH1* has been found to induce ferroptosis through ferritinophagy, a type of autophagy that involves ferroptosis to degrade ferritin (Tian et al., 2020). This finding suggests that FTH1 could be a novel potential pharmacological target for PD. Notably, FTH1 is secreted by oligodendrocytes in extracellular vesicles, and disruption of ferritin heavy chain release or expression in oligodendrocytes may lead to iron-mediated ferroptotic axonal damage (Mukherjee et al., 2020). Therefore, we hypothesize that *FTH1* may also be involved in the development of MSA. However, additional evidence for the remaining candidate ferroptosis-related genes associated with MSA-C is lacking, and further studies are necessary to elucidate the underlying mechanisms.

The connection between ferroptosis and immune infiltration is an important aspect that needs further elucidation. Understanding the interplay between these two processes is crucial for comprehending the pathogenesis of neurodegenerative diseases. In the context of MSA-C, we revealed the significant correlation between our ferroptosis-related candidate gene expression and immune cells. One proposed link between ferroptosis and immune infiltration involves the release of damage-associated molecular patterns during the process of cell death, which act as danger signals to activate immune cells and promote immune infiltration (Proneth and Conrad, 2019). It is important to note that the relationship between ferroptosis and immune infiltration may be bidirectional, with each process potentially influencing the other. Future research aimed at comprehensively exploring the interplay between immune responses and ferroptosis is important. Addressing this question is crucial for a better understanding of the disease mechanism and identifying potential therapeutic strategies.

The role of the immune system in the development of MSA-C has long been poorly understood. Gong et al. conducted research detecting altered peripheral immune traits in a cohort comprising both MSA-P and MSA-C patients, providing valuable evidence for peripheral immune dysregulation in these individuals (Gong et al., 2023). Additionally, Williams et al. (2020) discovered a significant increase in the number of CD3^+^ T cells, CD4^+^ T cells, and CD8^+^ T cells in postmortem brain sections of the putamen and substantia nigra in MSA patients compared to controls, suggesting T-cell infiltration in MSA. Further research has shown that the targeted elimination of αβ T cells or CD4^+^ T cells individually results in a reduction of central nervous system myeloid activation and demyelination in response to α-synuclein expression. These results suggest that T cells may play a critical role in mediating α-synuclein-induced inflammation and disease-causing demyelination (Williams et al., 2020). We also observed the infiltration of CD8^+^ T cells in the cerebellar white matter of MSA-C. T-cell priming and infiltration may be involved in the pathophysiological mechanism of MSA. It is hypothesized that chronic neuroinflammation resulting from immune activation may lead to neuronal and oligodendroglial cell death, thereby contributing to the neurodegenerative process observed in MSA.

The limitations of this study may include the following aspects. First, due to resource constraints, data of bulk RNA sequencing were used to identify global changes in gene expression patterns in this study. Further single-cell RNA sequencing may allow for the profiling of gene expression at the single-cell level, providing insights into cell heterogeneity and identifying cell-specific responses. Second, it is important to note that the sample size in our study is relatively small. A larger, well-balanced sample size is needed in further studies. Third, this study is based on a comprehensive bioinformatic analysis of available data, and experimental validation is a critical next step in confirming the mechanistic links we have proposed. Overall, the involvement of iron dysregulation and the immune system in the pathogenesis of MSA highlights the importance of considering ferroptosis and neuroinflammation in the development of potential therapeutic strategies. Further research is needed to fully elucidate the role of ferroptosis and the immune system in MSA and identify potential targets for therapeutic intervention.

## Data availability statement

The datasets presented in this study can be found in online repositories. The names of the repository/repositories and accession number(s) can be found in the article/supplementary material.

## Ethics statement

The study was approved by the Ethics Committee of Taizhou Central Hospital (Taizhou University Hospital). Patient consent was not required as this study was based on publicly available data.

## Author contributions

LC: Conceptualization, Writing—original draft, Writing—review & editing, Validation, Visualization. LM: Project administration, Writing—review & editing. HL: Validation, Writing—review & editing, Methodology, Supervision. PL: Methodology, Writing—review & editing, Conceptualization, Data curation, Formal analysis, Investigation, Writing—original draft.
